# GNX-4728, a novel small molecule drug inhibitor of mitochondrial permeability transition, is therapeutic in a mouse model of amyotrophic lateral sclerosis

**DOI:** 10.3389/fncel.2014.00433

**Published:** 2014-12-19

**Authors:** Lee J. Martin, Daniele Fancelli, Margaret Wong, Mark Niedzwiecki, Marco Ballarini, Simon Plyte, Qing Chang

**Affiliations:** ^1^Department of Pathology, Division of Neuropathology, Johns Hopkins University School of MedicineBaltimore, MD, USA; ^2^Pathobiology Graduate Program, Johns Hopkins University School of MedicineBaltimore, MD, USA; ^3^Department of Neuroscience, Johns Hopkins University School of MedicineBaltimore, MD, USA; ^4^Congenia Srl-Genextra GroupMilan, Italy

**Keywords:** motor neuron disease, therapeutics, motoneuron, mitochondrial permeability transition pore, mitochondria, mitochondrial calcium uptake, neuromuscular junction

## Abstract

Amyotrophic lateral sclerosis (ALS) is a fatal neurological disorder in humans characterized by progressive degeneration of skeletal muscle and motor neurons in spinal cord, brainstem, and cerebral cortex causing skeletal muscle paralysis, respiratory insufficiency, and death. There are no cures or effective treatments for ALS. ALS can be inherited, but most cases are not associated with a family history of the disease. Mitochondria have been implicated in the pathogenesis but definitive proof of causal mechanisms is lacking. Identification of new clinically translatable disease mechanism-based molecular targets and small molecule drug candidates are needed for ALS patients. We tested the hypothesis in an animal model that drug modulation of the mitochondrial permeability transition pore (mPTP) is therapeutic in ALS. A prospective randomized placebo-controlled drug trial was done in a transgenic (tg) mouse model of ALS. We explored GNX-4728 as a therapeutic drug. GNX-4728 inhibits mPTP opening as evidenced by increased mitochondrial calcium retention capacity (CRC) both *in vitro* and *in vivo*. Chronic systemic treatment of G37R-human mutant superoxide dismutase-1 (hSOD1) tg mice with GNX-4728 resulted in major therapeutic benefits. GNX-4728 slowed disease progression and significantly improved motor function. The survival of ALS mice was increased significantly by GNX-4728 treatment as evidence by a nearly 2-fold extension of lifespan (360 days–750 days). GNX-4728 protected against motor neuron degeneration and mitochondrial degeneration, attenuated spinal cord inflammation, and preserved neuromuscular junction (NMJ) innervation in the diaphragm in ALS mice. This work demonstrates that a mPTP-acting drug has major disease-modifying efficacy in a preclinical mouse model of ALS and establishes mitochondrial calcium retention, and indirectly the mPTP, as targets for ALS drug development.

## Introduction

Mitochondrial mechanisms have been implicated in the pathogenesis and progression of amyotrophic lateral sclerosis (ALS; Wong et al., 1995; Bendotti et al., 2001; Beal, 2005; Martin, 2010a; Reddy and Reddy, 2011; Muyderman and Chen, 2014). Mitochondrial-based mechanisms of disease in ALS might include failure of intracellular Ca^2+^ homeostasis, oxidative stress propagation, energy depletion, perturbed fission-fusion dynamics, and cell death initiation (Beal, 2005; Reddy and Reddy, 2011), but it is possible that mitochondrial changes are indirectly related to disease etiology and are secondary or bystander events (Morais and De Strooper, 2010; Muyderman and Chen, 2014). Previous therapeutic studies have approached the mitochondrial role in ALS using antioxidants, metabolic boosters, and dietary manipulations, mostly in mouse models (Reddy and Reddy, 2011), but these approaches generally lacked specificity for establishing mitochondria or specific components or properties of mitochondria as disease targets. Recently, drugs with putative mitochondrial mechanisms of action, such as dexpramipexole and olesoxime, have failed in human ALS clinical trials (Cudkowicz et al., 2013; Lenglet et al., 2014), but these drugs have pleiotropic actions or unclear mitochondrial mechanisms of action. Well-defined, unequivocal and specific mitochondrial mechanisms and targets for directed therapeutics and disease prevention in ALS have remained elusive.

The mitochondrial permeability transition pore (mPTP) is emerging as a critical player in neurodegenerative disease and in acute neuropathology (Martin et al., 2009; Martin, 2010a,b). While the definitive core components of the mPTP have been fleeting (Bernardi et al., 2006; Halestrap, 2009), but now thought to involve the c-subunit ring of the F_1_F_0_ ATP synthase (Bonora et al., 2013; Alavian et al., 2014), a consistent critical regulator of the mPTP *in vivo* and *in vitro* is cyclophilin D (Bernardi et al., 2006; Halestrap, 2009; Alavian et al., 2014). A cyclophilin D knockout study was important in establishing mitochondria as having a direct role in the mechanisms of disease in preclinical mouse models of ALS (Martin et al., 2009). The mPTP as a target of therapeutics in ALS (Martin, 2010b) needs to be validated and then translated to preclinical animal models using meaningful pharmacologic approaches rather than genetic approaches. Very few drugs have been validated as compounds specifically targeting putative components or functions of the mPTP such as CRC. A class of cinnamic anilide derivatives has been recently synthesized and identified as mPTP inhibitors endowed with *in vivo* therapeutic activity in protecting heart mitochondria from calcium overload and rabbit heart from ischemia (Fancelli et al., 2014). These compounds are able to inhibit mPTP opening in response to calcium overload, oxidative stress, and chemical cross-linkers in isolated mitochondria (Fancelli et al., 2014). We studied GNX-4728, a cinnamic anilide compound from the same series, which inhibits the mPTP and protects mitochondria from calcium overload by increasing CRC. We then tested GNX-4728 for therapeutic actions in a transgenic (tg) mouse model of ALS. This study shows that chronic treatment of G37R-human mutant superoxide dismutase-1 (hSOD1) tg mice with GNX-4728 strongly protects against onset of ALS and robustly extends survival with preservation of motor neuron number, motor neuron mitochondria, and neuromuscular junction (NMJ) integrity.

## Materials and methods

### Mice

Adult wildtype non-tg C57BL/6 mice and tg mice were used. Tg mice were hemizygous for a low copy number of hSOD1-G37R mutant allele driven by the endogenous human promoter (line 29) derived from a founder B6.Cg-Tg SOD1-G37R 29Dpr/J (stock # 008229, The Jackson Laboratory, Bar Harbor, MA) as described (Gertz et al., 2012; Wong et al., 2013). Mice were used with approval from the institutional Animal Care and Use Committee.

### Drug

**Figure 1:**
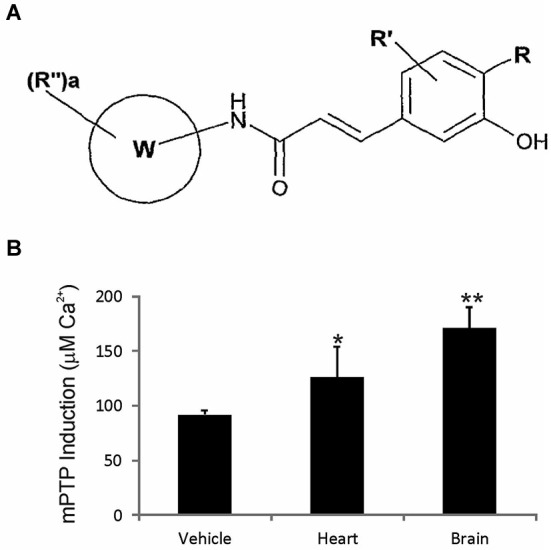
**GNX-4728 general structure and *in vivo* actions on mitochondria. (A)** General structure of the chemical class of cinnamic anilide mPTP inhibitors that comprises GNX-4728. **(B)** Organ (heart and brain) calcium retention capacity (CRC) assay performed on freshly prepared mitochondria following systemic treatment of mice with GNX-4728 or vehicle. CRC was determined by the concentration of calcium required to trigger mPTP opening. CRC was increased by GNX-4728 in heart (*p* < 0.05) and brain (*p* < 0.01) compared to vehicle (combined organ mitochondria).

GNX-4728 is a substituted cinnamic anilide (Figure 1A) which belongs to a novel series of potent inhibitors of the mPTP (Fancelli et al., 2014).

### Mitochondrial calcium retention capacity (CRC) assay

CRC assays were performed on freshly isolated mitochondria from adult non-tg mouse brain and heart (*n* = 6) after GNX-4728 was administered intravenously by tail vein injection (15 mg/kg in 20% DMSO and 40% PEG400) followed by a survival of 5 min. Control mice (*n* = 6) were injected with vehicle. Brain and heart mitochondria were isolated using a similar procedure as described (Wong et al., 2013). Mitochondrial CRC was assessed fluorimetrically in the presence of the fluorescent Ca^2+^ indicator Calcium Green 5N (Invitrogen Molecular Probes) using a temperature controlled Perkin-Elmer LS 55 spectrofluorimeter as described (Fancelli et al., 2014). Briefly, purified organ mitochondria were pulse-loaded with 10 mM calcium and then challenged with increasing concentrations of calcium until mitochondrial permeability transition was triggered as evidenced by complete release of mitochondrially-stored calcium due to mPTP opening.

### Tg mice and drug treatment protocol

Cohorts of tg mice expressing mutated G37R-hSOD1 were bred and identified by genotyping of tail DNA as described (Martin et al., 2007, 2009; Wong and Martin, 2010). All mice were housed in the institutional vivarium with generally 4–5 mice per cage and *ad libitum* food and water. Starting at 6 months of age, before the onset of overt symptoms, male G37R-hSOD1 mice were treated with 300 µg (100 µl) of GNX-4728 or vehicle (DMSO/cyclodextrin/saline) every other day by intraperitoneal injection. Only male mice were used because of known gender-differences in the involvement of the mPTP regulator cyclophilin D in ALS pathobiology (Martin et al., 2009) and to minimize burden to the operators treating mice over long-term with individual injections. This dosage and treatment regimen was in part based on preliminary data showing GNX-4728 protection of ALS mouse spinal cord mitochondria from Ca^2+^-induced swelling (Martin et al., unpublished observations). The treatment group size totals were 18–20 mice, but these mice were divided among different experiments, including survival studies. The initial randomization was accomplished using the envelope method with knowledge of litter origin so that individuals from the same litter could be distributed across groups. Mice were maintained in the colony and received vehicle or GNX-4728 treatment. For survival experiments, the vehicle and GNX-4728 group sizes were 10 and 12, respectively. The mice were observed once a day. The disease progresses usually from hindleg paraplegia with some function of the forelimbs remaining. When animals developed paraplegia, *ad libitum* nutrigel and chow were placed in the cage, and water was available at a bottom-cage level drinking spout. At this time locomotor activity is compromised but the animals can still ambulate to access food and water. At this stage, the mice are observed three or more times a day. Endstage disease was defined as complete lack of locomotor activity, detected within 2–4 h after onset, at which time the mice were euthanized. A subgroup of mice was killed before endstage to assess the efficacy of GNX-4728 using histological endpoints. Disease onset was assessed by running wheel activity and hind-limb paresis. Vehicle and GNX-4728 treated mice and age-matched non-tg littermate mice were evaluated for neurologic deficit. They were assessed at 12 months of age using a voluntary activity wheel (Harvard Apparatus).

### Spinal cord and neuromuscular pathology

Vehicle and GNX-4728 treated tg mice and naïve mice (*n* = 5 per group) at 12 months of age were deeply anesthetized and perfused by cardiac puncture with ice-cold 100 mM phosphate-buffered normal saline (PBS) followed by 4% paraformaldehyde in PBS. After perfusion-fixation, mouse bodies were stored at 4°C overnight and then the spinal cord and diaphragm were removed from each mouse. Spinal cords were cryoprotected (20% glycerol) before they were frozen-sectioned (40 µm) transversely using a sliding microtome. Serial tissue section arrays were stored individually in 96-well plates in anti-freeze buffer. The diaphragm was removed as a complete tissue sheet (Comerford and Fitzgerald, 1986) and placed in PBS at 4°C until processed for NMJ visualization.

Nissl-stained transverse sections of spinal cord were used to count the number of motor neurons in vehicle and GNX-4728 treated tg mice and in age-matched littermate non-tg mice. Spinal cord sections were selected with a random start and then systematically sampled (every 10^th^ section) to generate a subsample of sections from each mouse lumbar spinal cord that was mounted on glass slides and stained with cresyl violet for cell counting. Nissl-stained motor neurons in ventral horn were counted by individuals blinded to experimental treatment, using strict morphological criteria, in digital images acquired with a Nikon microscope at 200x magnification. These criteria included a round, open, pale nucleus (not condensed and darkly stained), globular Nissl staining of the cytoplasm, and a diameter of ~20–40 µm. With these criteria, astrocytes, oligodendrocytes, and microglia were excluded from the counts, but these counts are likely to estimate the combined populations of α- and γ-motor neurons.

Mitochondrial pathology was assessed specifically within spinal cord motor neurons using immunohistochemistry. Free-floating spinal cord sections from GNX-4728- and vehicle-treated G37R-hSOD1 tg mice and age-matched non-tg mice were stained by an immunoperoxidase method with antibodies to the mitochondrial matrix marker superoxide dismutase-2 (Stressgen) and diaminobenzidine as described (Martin et al., 2007). Mitochondrial diameters within motor neurons were measured by individuals blinded to experimental history using ocular filar micrometry as described (Martin et al., 2007).

Diaphragm motor endplates were visualized with Alexa 594-conjugated α-bungarotoxin (BTX, Invitrogen, Molecular Probes) as described (Martin and Liu, 2007). Dual labeling was done to visualize motor neuron distal axons and their terminals in whole diaphragm preparations by immunofluorescent detection of neurofilament protein using a monoclonal antibody (SMI-32, Convance) and confocal microscopy as described (Martin and Liu, 2007). The immunofluorescent labeling for neurofilament was used to determine whether the BTX-labeled motor endplates were innervated. Confocal microscope images of the typical band distributions of motor endplates in diaphragm (Comerford and Fitzgerald, 1986) were scored as innervated (normal) if there was overlap with the axon terminal or denervated (unoccupied) if the endplate was not associated with an axon. NMJ imaging and scoring were performed by individuals unaware of mouse treatment.

### Data analysis

The values shown in the graphs represent the mean ± standard deviation. For histological data, group means and variances were evaluated statistically by one-way ANOVA and a Student’s *t*-test. Time-to-event measures (disease onset and survival duration) were analyzed using Kaplan-Meier survival fit analysis. The Cox proportional hazards model was used to analyze the effect of GNX-4728 on survival and to determine hazard ratios. There was no censoring of mice due to drug- or treatment-related deaths. A one-way ANOVA followed by Tukey *post-hoc* test were used for statistical comparisons for time-to-event measures.

### Photography and figure construction

The original images used for figure construction were generated using digital photography. Digital images were captured as TiFF files using a SPOT digital camera and SPOT Advanced software (Diagnostic Instruments) or a Nikon digital camera (DXM1200) and ACT-1 software. Images were altered slightly for brightness and contrast using ArcSoft PhotoStudio 2000 or Adobe Photoshop software without changing the content and actual result. Figure composition was done using CorelDraw X5 software with final figures being converted to TiFF files. Files of composite figures were adjusted for brightness and contrast in Adobe Photoshop.

## Results

### GNX-4728 enhances mitochondrial CRC

The mPTP blocking activity and blood brain barrier permeability of GNX-4728 *in vivo* was assessed by measuring its ability to prevent isolated calcium pulse-loaded organ mitochondria from mPTP opening after systemic drug treatment. Mice were treated with 15 mg/kg GNX-4728 or vehicle iv and, 5 min later, mitochondria were isolated from heart and brain and then assayed for mPTP opening induced by calcium (Figure 1B). Systemic treatment with GNX-4728 significantly increased mitochondrial CRC in heart and brain (Figure 1B). The increase in brain mitochondrial CRC is indicative of the ability of GNX-4728 to cross the blood-brain barrier (BBB). Other compounds of this series were able to protect heart, liver and kidney, but not brain mitochondria after *in vivo* administration which supports the notion that GNX-4728 actively crosses the BBB (data not shown).

### GNX-4728 increases lifespan of ALS mice

**Figure 2:**
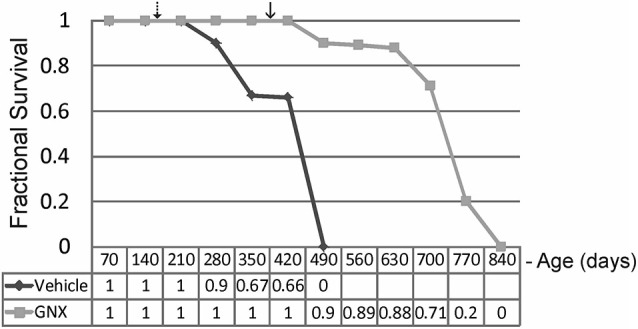
**Chronic treatment of G37R-human mutant superoxide dismutase (hSOD1) transgenic mice with GNX-4728 is therapeutic in ALS**. Kaplan-Meier survival plot for age at death in G37R-hSOD1 mice. GNX-4728 treatment of G37R-hSOD1 mice (*n* = 12) robustly extended survival compared to littermate vehicle-treated ALS mice (*n* = 10). Disease onsets for vehicle-treated (hatched arrow) and GNX-treated (solid arrow) are indicated. See text for description of hazards ratios (at graph bottom).

GNX-4728 treatment of G37R-hSOD1 mice strongly protected against clinical onset of disease and robustly extended survival compared to littermate vehicle-treated ALS mice (Figure 2). Disease onset (mean ± SD), as assessed by running wheel activity and hind-limb paresis, was significantly (*p* < 0.01) delayed with GNX treatment (381 ± 45 days) vs. vehicle (159 ± 39 days). Median lifespan (mean ± SD) was significantly (*p* < 0.01) increased with GNX treatment (686 ± 120 days) vs. vehicle (366 ± 29 days). Hazard ratio determinations of the two treatment groups revealed significantly different hazard rates for the GNX-4728 treated mice as 0, 0.1, 0.11, 0.12, 0.29, 0.8, and 1.0 at 420, 490, 560, 630, 700, 770, and 840 days of age, respectively (Figure 2). For some ALS mice treated with GNX-4728 their lifespan was almost doubled (Figure 2).

### GNX-4728 protects against spinal cord, mitochondrial, and diaphragm pathology in ALS mice

**Figure 3:**
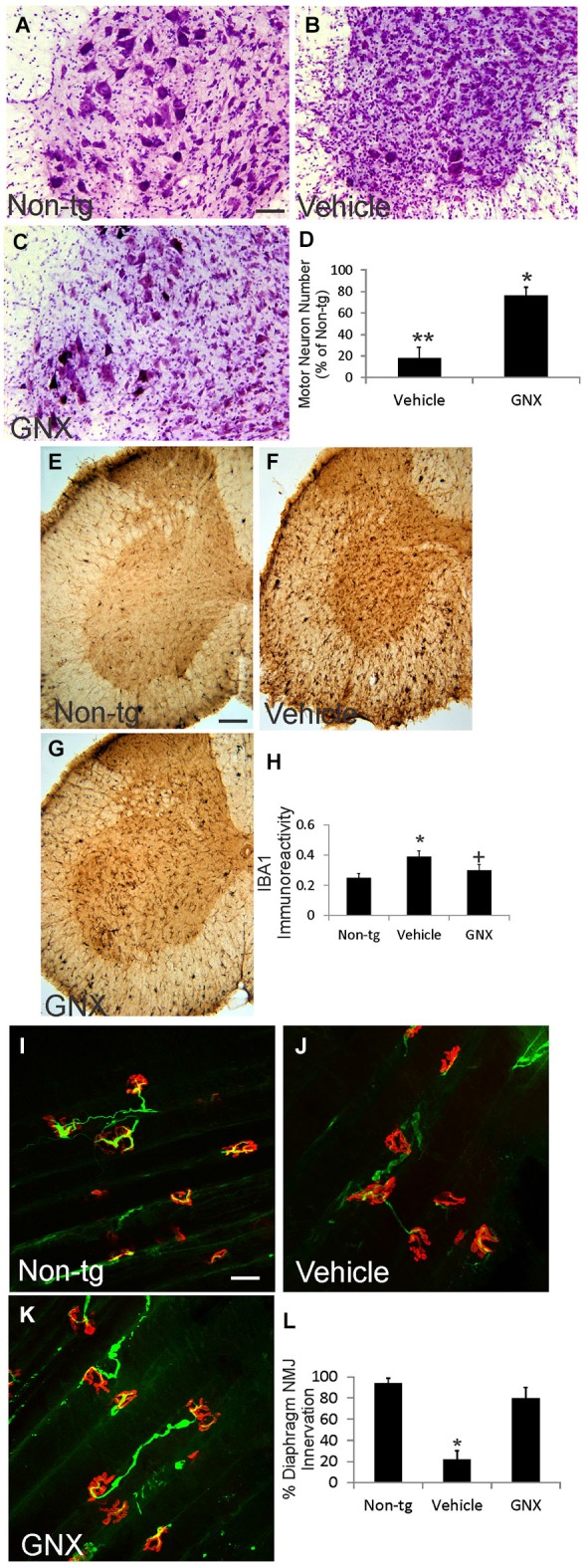
**GNX-4728 protects spinal cord motor neurons and diaphragm neuromuscular junctions (NMJs) in G37R-hSOD1 tg mice**. **(A–C)** Brightfield microscope images of cresyl violet (Nissl)-stained lumbar spinal cord sections from a 12-month-old non-tg mouse **(A)** and G37R-hSOD1 tg mice that received vehicle **(B)** or GNX-4728 **(C)** treatments. Scale bar (in **A**) = 40 µm (same for **B,C**). **(D)** Graph showing the number of lumbar spinal cord motor neurons in G37R-hSOD1 tg mice that received vehicle or GNX-4728 (GNX) treatments. Values are mean ± SD (*N* = 5/group). Significant differences **p < 0.001 or *p < 0.05 from non-tg mice. **(E–G)** Brightfield microscope images of Iba1 (microglial marker)-immunostained lumbar spinal cord sections from a 12-month-old non-tg mouse **(E)** and G37R-hSOD1 tg mice that received vehicle **(F)** or GNX-4728 **(G)** treatments. Scale bar (in **E**) = 100 µm (same for **F,G**). **(H)** Graph showing the immunodensity of spinal cord Iba1 immunoreactivity in non-tg mice and G37R-hSOD1 tg mice that received vehicle or GNX-4728 (GNX) treatments. Values are mean ± SD (*N* = 5/group): significant difference *p < 0.05 from non-tg mice; significant difference ^+^*p* < 0.05 from vehicle-treated mice. **(I–K)** Confocal microscope images of diaphragm NMJs stained for skeletal muscle motor endplates (α-bungarotoxin, red) and motor neuron axons (neurofilament, green) from a 12-month-old non-tg mouse **(I)** and G37R-hSOD1 tg mice that received vehicle **(J)** or GNX-4728 **(K)** treatments. Scale bar (in **I**) = 100 µm (same for **J,K**). **(L)** Graph showing the percent innervation of motor endplates in diaphragm. Significant difference *p < 0.01 from non-tg mice.

Twelve-month-old vehicle-treated and GNX-4728-treated ALS mice and age-matched non-tg mice were evaluated histologically for motor neuron numbers, mitochondrial swelling, and inflammatory changes in spinal cord and for NMJ innervations in diaphragm (Figure 3; Table 1). Nissl-staining was used to visualize motor neurons in spinal cord (Figures 3A–C). In preparations of non-tg mice, large spinal motor neurons were prominent and inflammatory changes were not present (Figure 3A). At 12 months of age, vehicle-treated G37R-SOD1 tg mice were symptomatic and had about 80% loss of motor neurons in lumbar spinal cord (Figures 3B,D) and prominent small cell infiltration and reactive inflammatory changes (Figure 3B). Iba1 immunoreactivity, a marker for inflammation (Zhao et al., 2013), was significantly elevated in vehicle-treated ALS mouse spinal cord compared to non-tg control (Figure 3A). In contrast, 12-month-old GNX-4728-treated ALS mice had significant preservation of motor neurons (Figures 3C,D) and significantly attenuated inflammation as evidenced by the diminished Iba1 immunoreactivity (Figures 3F–H).

**Table 1:** **GNX-4728 protection of spinal cord motor neuron mitochondria**.

Group	Mitochondrial diameter (µm)^1^
Non-tg	0.4 ± 0.2
G37R-hSOD1 Vehicle	1.6 ± 0.6**
G37R-hSOD1 GNX-4728	0.7 ± 0.4*

^1^Values are mean ± standard deviation. *GNX4728 vs. non-tg and vehicle, *p* < 0.05. **Vehicle vs. non-tg, *p* < 0.01.

To assess the ability of GNX-4728 to protect mitochondria directly within motor neurons we used immunohistochemistry to detect the mitochondrial marker SOD2 and measured mitochondrial diameters. In non-tg mouse spinal motor mitochondria are about 0.4–0.5 µm in diameter (Table 1), consistent with previous observations (Martin et al., 2007). In 12-month-old vehicle-treated G37R-hSOD1 tg mice, mitochondrial diameters were increased significantly compared to age-matched non-tg mice (Table 1). GNX-4728 significantly protected against mitochondrial swelling in G37R-hSOD1 tg mouse spinal motor neurons (Table 1).

To assay for whether GNX-4728 protects NMJs in ALS mice, a whole-mount diaphragm preparation was used. In non-tg mice, motor endplate innervation of diaphragm was near 100% (Figures 3I,L), while in vehicle-treated ALS mice endplate innervation was only about 20% (Figures 3J,L). In contrast, in GNX-4728-treated ALS mice, NMJ innervation was restored to about 80% of non-tg control (Figures 3K,L).

## Discussion

Our study demonstrates that a small molecule cinnamic anilide derivative, GNX-4728, has several major therapeutic benefits in a mouse model of ALS. GNX-4728 increased brain mitochondrial CRC *in vivo* after systemic administration. Importantly from a preclinical perspective, chronic systemic treatment of ALS mice with GNX-4728 resulted in the following: (1) a delay in disease onset; (2) dramatically increased lifespan; (3) protection of spinal cord motor neurons; (4) protection of spinal cord motor neuron mitochondria; (5) block of spinal cord inflammatory changes; and (6) preservation of NMJs in diaphragm. These results are particularly exciting because they support the concept of the mPTP as a practical drugable therapeutic target in ALS *in vivo* and demonstrate that cinnamic anilides could be a future avenue to the effective treatment of ALS.

In this study we used a tg mouse model of ALS to test the therapeutic efficacy of GNX-4728. These mice express a low copy number of hSOD1-G37R mutant allele in a non-conditional expression pattern throughout the body (Wong et al., 1995). This mouse model is very different from the hSOD1-G93A tg mouse model that expresses the mutant allele at a very high copy number and consequently have an aggressive disease and a lifespan of only about 4 months (Gurney et al., 1994). Because of the rapidity of the disease course, the hSOD1-G93A high-expresser tg mouse has been the most commonly used mouse model to assess ALS therapeutics. However, although these mice develop a fatal paralysis and show a very prominent lower motor neuron disease (Gurney et al., 1994; Bendotti et al., 2001; Martin et al., 2007), the details of the cellular and molecular pathology appear to be distinct from that seen in human ALS (Martin, 1999, 2010a; Martin and Liu, 2004; Martin et al., 2007). These differences might contribute to the lack of success in the clinical translation of drugs shown to have therapeutic efficacy in the hSOD1-G93A tg mouse model. Therefore, we used, instead of the high-expressing hSOD1-G93A tg mouse line, the hSOD1-G37R mouse line with a much longer lifespan. The major downsides of using the longer-lived mouse line is the time commitment for drug treatments and the modeling of only a subtype of familial ALS caused by a mutated hSOD1 gene.

We found that GNX-4728 improved neurologic function and survival of ALS mice. The effect on lifespan was robust. A limitation of the current study is the relative small group sizes for the survival analysis, as it would have been better to have doubled the number of animals in each group. Few pharmacological studies have been done using the hSOD1-G37R mouse line, and the most notable study showed that mice fed chow containing a cocktail of riluzole, nimodipine, and minocycline had about a 15% increase in maximal lifespan (Kriz et al., 2003). This modest effect might be due to the lack of direct disease mechanism-based targeting. In fact, treatment of human ALS patients with riluzole, a sodium channel antagonist with anti-glutamatergic actions, prolongs life by only about 2–3 months in some patients, but there is no visible functional improvement (Bensimon et al., 1994). Questions persist about the clinical usefulness of riluzole because of its modest effects and high cost (Miller et al., 2012). Another anti-glutamate drug, gabapentin, failed in clinical trials (Miller et al., 2001). Thus, a role for glutamate and excitotoxicity in the direct mechanisms of ALS is appearing tenuous (Martin, 2010a), and the concept has so far failed to bear effective therapeutic fruit after more than 20 years (Rothstein et al., 1992). Other disease-mechanism concepts need to be considered to potentially move the treatment of ALS forward. A role for the mPTP in the mechanism of ALS is an attractive concept that can bring together old and new ideas regarding cell degeneration in ALS including, intracellular calcium dysregulation, mitochondria, oxidative stress, and possibly excitotoxicity (Martin, 2010a,b; Reddy and Reddy, 2011). In the current preclinical animal model study, we attribute the success of GNX-4728 to its actions on mitochondria. It penetrated the BBB and increased mitochondrial CRC in brain, revealed by the increased threshold for calcium-induced permeability transition, suggesting that GNX-4728 is operating by inhibiting the mPTP. Striated muscle mitochondrial CRC was also increased. Genetic studies have revealed the participation of the mPTP in the mechanisms of disease in mouse models of ALS (Martin et al., 2009). Other studies support the role of mitochondrial calcium dysregulation and mPTP activation in the mechanisms of ALS (Bendotti et al., 2001; Jaiswal and Keller, 2009; Barrientos et al., 2011; Nguyen et al., 2011; Bartolome et al., 2013; Muyderman and Chen, 2014). The finding that GNX-4728 bocks mitochondrial swelling directly within motor neurons and protects these cells further points to the mPTP as a target of disease in ALS and that GNX-4728 is possibly providing therapeutic benefit through modulation of mPTP function.

GNX-4728 was delivered systemically in our study. Its therapeutic activity could be within the CNS, because of its BBB permeability, at peripheral locations (skeletal muscle and nerves), or both. It could be acting at skeletal muscle mitochondria to provide therapeutic effects. Skeletal muscle is also a primary site of disease in mouse models of ALS (Dobrowolny et al., 2008; Wong and Martin, 2010; Luo et al., 2013) and possibly in human ALS (Vielhaber et al., 2000). Mitochondria within skeletal muscle of ALS mice show early abnormalities in calcium signaling capacity (Zhou et al., 2010), DNA methylation and autophagy (Wong et al., 2013), and metabolism (Dupuis et al., 2006). Additional studies need to address the potential effects of GNX-4728 and non-BBB permeable cinnamic anilides in skeletal muscle of ALS mice.

Despite evidence derived from human ALS and mouse and cells models of ALS indicating that mitochondria have a role in disease pathogenesis (Beal, 2005; Martin, 2010a; Reddy and Reddy, 2011), recent human ALS clinical trials with putative mitochondrial acting drugs have been unsuccessful. Dexpramipexole and olesoxime both failed to show significant benefits in ALS patients (Cudkowicz et al., 2013; Lenglet et al., 2014). Dexpramipexole can act on brain mitochondria to increase the efficiency of oxidative phosphorylation (Alavian et al., 2012). Olesoxime has pleiotropic actions. It protects neurons from trophic factor and target deprivation and apoptosis (Bordet et al., 2007; Martin et al., 2011). Olesoxime binds proteins in the outer mitochondrial membrane (Bordet et al., 2007, 2010), but it does not increase mitochondrial CRC (Bordet et al., 2010). Thus, the failure of dexpramipexole and olesoxime as therapies for human ALS could be due to their inability to modulate mitochondrial CRC.

To our knowledge, no other experimental drug or experimental manipulation has shown this level of therapeutic efficacy in ALS mice. We have not observed untoward side-effects *in vivo*, even after chronic treatment of mice. This study identifies a new drug class, cinnamic anilides, which might be promising for the treatment of ALS. Future experiments are warranted that test the efficacy of GNX-4728-like compounds in other mouse models of ALS to assess their potential general application for the treatment of different forms of ALS.

## Author contributions

Conceived and designed experiments: Lee Martin, Simon Plyte. Provided reagents: Daniele Fancelli, Simon Plyte. Performed experiments: Lee Martin, Margaret Wong, Marco Ballarini, Qing Chang. Analyzed the data: Lee Martin, Margaret Wong, Qing Chang, Mark Niedzwiecki. Wrote the paper: Lee Martin, Daniele Fancelli, Simon Plyte.

## Conflict of interest statement

The study was funded in part by Congenia Srl-Genextra.
